# Development of a novel approach for construction of high gradient braze-free S-band cavities

**DOI:** 10.1038/s41598-021-98238-8

**Published:** 2021-09-22

**Authors:** Mahdi Aghayan, S. Farhad Masoudi, Farshad Ghasemi, Walter Wuensch, Hamed Shaker

**Affiliations:** 1 Department of Physics, K.N. Toosi University of Technology, P.O. Box 15875-4416, Tehran, Iran; 2 Physics and Particle Accelerators Research School, Nuclear Science and Technology Research Institute, Tehran, Iran; 3 CERN, European Organization for Nuclear Research, Geneva, Switzerland; 4 Canadian Light Source, Saskatoon, Canada

**Keywords:** Experimental nuclear physics, Design, synthesis and processing, Physics

## Abstract

Vacuum breakdown is one of the main limitations to the operating accelerating gradient in radio frequency linear accelerators. Recent studies of copper cavities have been shown that harder copper conditions more quickly and can reach higher accelerating gradients than soft copper cavities. Exploiting this advantage requires the development of assembly methods that do not involve the copper-softening high-temperature heating cycles that are used in for example bonding and brazing. A shrink-fit method, which was already implemented successfully in the operation the IPM linac, is proposed for the construction high-gradient test S-band standing wave structure operating at 2998.5 MHz. The three cells cavity is designed to have a maximum gradient in the middle cell that is twice that of the adjacent cells. Mechanical considerations relating to the shrink-fit construction method have been performed using Ansys. To validate the simulations and ensure the feasibility of construction by shrink-fit method, a sample cavity was constructed and cold tests was performed.

## Introduction

The electron linear accelerator (linac) is one of the most widely used accelerator in the world and has a broad range of applications including radiotherapy, industrial irradiation, cargo scanning, beam injection to high-energy accelerators, particle physics and free electron lasers. Reducing linac size and cost is important for all these applications and a priority for accelerator scientists and engineers. Because accelerating gradient determines the length of the accelerator for a fixed final energy, high-gradient accelerator technology can minimize linac length. This can result in more compact and lower cost facilities, expanding the range of applications of linacs.

In recent years, research on high-gradient accelerators has become one of the most exciting topics for accelerator research groups. The different aspects of the research for high gradient cavities includes radio-frequency (RF) design^1–3^, materials^4,5^ and construction methods^6–9^. This report will describe innovations in construction methods supported by a comprehensive design methodology.

The most common methods used to join machinable single cells into the multi-cell structures needed for linacs are brazing and bonding, which are carried out in high-temperature furnaces. In the brazing method, two cells are joined together by melting a filler metal placed into the joint. This technique requires sophisticated procedures and equipment therefore it is a costly cavity construction method. Moreover, the high temperature heating cycle needed to melt the filler material in the brazing method, results in larger crystal grains and softens the copper. On the other hand, it has been shown that hard un-annealed copper-alloy material is better than softened copper desirable for achieving high accelerating gradients^4,6,10,11^. So non-brazing methods have been used for construction of high gradient structures.

A number of assembly techniques that do not involve heating to high-temperature have been developed. A new fabrication technique based on clamping has been recently developed and used for the construction of a high gradient S-band photocathode RF gun by INFN-LNF, Italy. The RF gun was successfully tested up to a field of 120 MV/m on the cathode surface^7,12^. Another clamping method, made in combination with electron beam welding (EBW) and Tungsten Inert Gas (TIG) welding, has been used at SLAC for the construction of a high gradient X-band single cell cavity^6,11^. A novel technique of assembling the structure from milled halves has been used for a prototype 11.994 GHz, traveling-wave accelerating structure for the Compact Linear Collider^3^. Reduction in cost, as well as a greater freedom in choice of joining techniques are advantages of half and quadrant-based technology and may provide a way to produce an accelerating structure of hard copper. The procurement and testing of such a hard copper prototype structure is underway in the context of the CLIC project^10^.

A 24-cell traveling-wave structure based on a quadrant-type fabrication method was built at KEK. EBW was used for joining the four quadrants. The measured RF characteristics were found to be reasonable even after welding. Also high-gradient tests was performed successfully for a single-cell standing-wave test cavity constructed with this method^13,14^.

This paper describes an alternative braze-free method for construction of high gradient cavities which is based on shrink-fitting. The cavity pieces are not heated to high temperature in this method, so the copper structure remains hard and is expected to perform better at high gradient fields. This method was used recently to fabricate and assemble S-band acceleration cavities of electron linear accelerator at the Institute for research in fundamental science (IPM-Iran) which was successfully tested up to 2 MW^15–19^.

In this paper, the design of a three cell S-band accelerating cavity is presented as well as a comprehensive analysis of issues arising from the shrink-fit method. The radio frequency design was made using high frequency structure simulator (HFSS, version 18.0, https://www.ansys.com) software. Electric and magnetic fields as well as the modified Poynting vector were calculated and employed to optimize the design. The mechanical deformations and stresses caused by shrink-fitting method were investigated, and the effect on resonant frequency was studied using Ansys software. To validate the simulations and ensure the feasibility of construction by shrink-fit method, an 8-cavity sample was constructed and cold tests were performed.

## Description of the shrink-fit method for the construction of high gradient cavities

Shrink fitting as a construction method for the assembly mechanical parts, is based on reducing the dimensions of metals at low temperatures. This method was used at SLAC for the MarkIII^20^ acceleration cavities but brazing was used for the two-mile Stanford linear accelerator. Brazing became the preferred method, shrink fitting no longer used and the technology has lain dormant until it has been recently reestablished at IPM. It was successfully used for a buncher and a 24-cell cavity at IPM. High-power tests reached 2 MW RF input power^18,19,21^ which demonstrates a good current carrying capacity of the joints. The critical issue is that joints in circular cavities like those used in the MarkIII and IPM structures need to carry RF currents. Interruptions to the current carrying path can result in lower Q factor and sparking at the joint.

The success of this method to construct and assemble the buncher and acceleration tube at IPM led to the suggestion of using it for high-gradient cavities. Subsequently a one meter long multi-cell accelerating structure with a gradient of 50 MV/m was planned. As a first step, the design and construction of a 3-cell S-band cavity has been carried out in order to evaluate the feasibility the shrink-fit method for high-gradient cavities.

Since the sensitivity to the quality of the joint is higher for a high-gradient cavity than a low gradient cavity, a comprehensive investigation the different aspects of the shrink-fit method was made, including thermomechanical and dimensional issues.

There are some challenges in the construction of a disc loaded waveguide cavity using the shrink-fit method, that need to be considered in the radio frequency and mechanical design stages. The components of the cavity are separated into internal and external parts. The internal parts (the disks) are cooled to liquid nitrogen temperature and shrink. At room temperature, the internal components dimensioned to have the same or slightly larger size than the external components (cylindrical waveguide). The internal parts must be dimensioned such that after cooling can be placed inside the external parts. After reaching ambient temperature, and expanding these parts will be fixed in place. Some of the important challenges which should be considered for assembling of cavities in this method are:

- accurate calculation of the part’s dimensions in different temperatures,
- investigation each disk pressure effect on wall
- design of a suitable fixture for maintaining internal components at precise distances which can be released easily after
- inserting internal parts in external parts without tilting and dislocating

**Figure 1:**
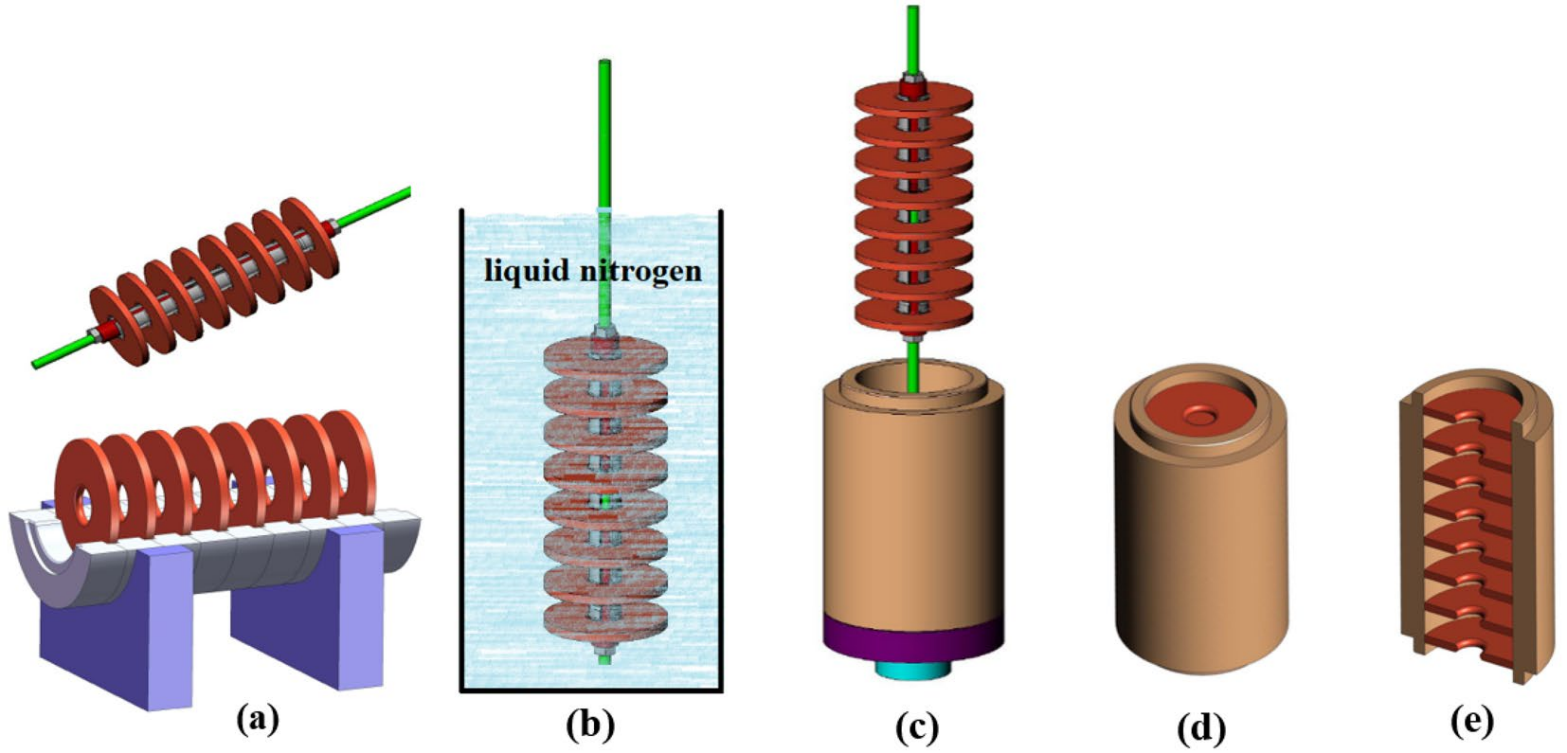
Assembly for a disk loaded cavity using shrink-fit method. (**a**) Fixture and fixing base which is used to hold the disks between certain and equal distance (**b**) shrinking of disks in liquid nitrogen temperature (**c**) disks are placed within the cylindrical waveguide (**d**). The discs gradually expand in ambient temperature and tighten in their place (**e**) inside final cavity.

Figure 1 shows the steps of assembly for a disk loaded cavity with this method.

## Radiofrequency design

The three cell cavity design is based on an X-band design that has been used for a program of studying different materials and construction methods of high gradient cavities at SLAC^4,22^. The design gives a high gradient cell located between the first and last lower field cells. The peak on-axis electric field in the middle cell is about two times higher than the surrounding cells. We used the same basic principle for the design of our S-band, 2998.5 MHz cavity but some changes have been required due to constraints given by the shrink-fit construction method. In particular, the radius of all cells was fixed to be equal to avoid steps on the inner surface of the cylindrical waveguide, which reduces the high gradient capability.

**Figure 2:**
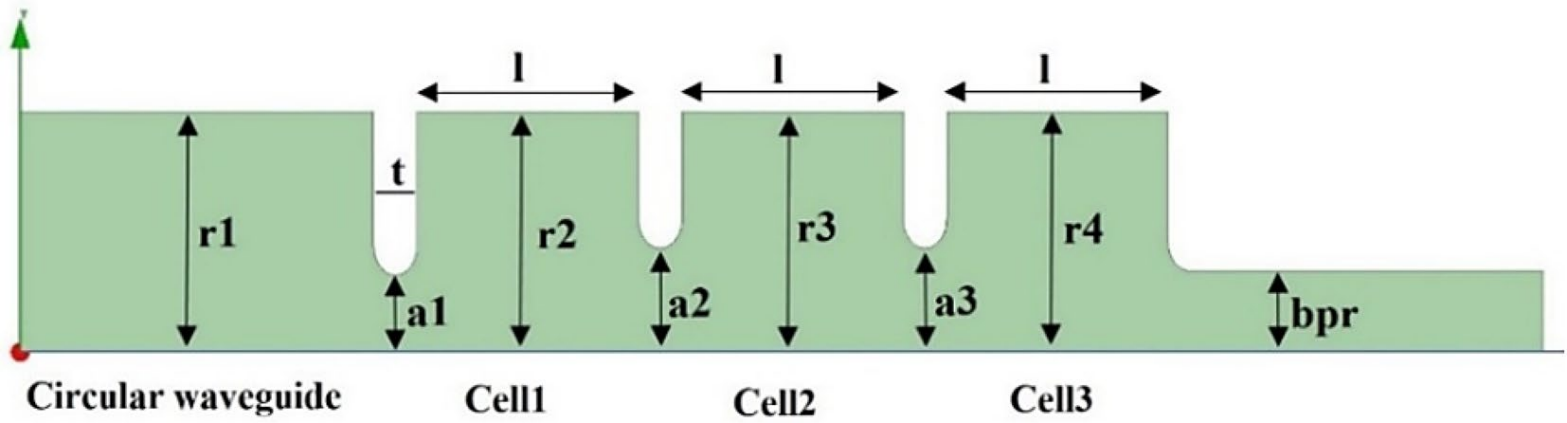
The structure of designed cavity.

Figure 2 shows the geometry of the cavity containing 3 cells, where a_1_ to a_3_ are the iris of the cells and r_1_ is the radius of cylindrical waveguide.

The goal of the RF design was to achieve an average gradient about 70 MV/m in the middle cell with breakdown rate (BDR) less than ${10}^{-6}\,{\mathrm{bpp}}/{\mathrm{m}}$ with an input power of 8 MW, which corresponds to the power from a Thales TH2157 Klystron. The modified Poynting vector (S_c_) parameter was used to maximize gradient for the target maximum breakdown rate. The equation for the modified Poynting vector is given in Eq. (1) where $\overline{S }$ is the complex Poynting vector^23^:

1 $$S_{c} = Re\left\{ {\overline{S}} \right\} + \frac{1}{6}Im\left\{ {\overline{S}} \right\}$$

Based on experimental data from multiple cavities, this value should not exceed $5\,{\mathrm{W}}/\upmu {\mathrm{m}}^{2}$ in order to have a BDR less than ${10}^{-6}\,{\mathrm{bpp}}/{\mathrm{m}}$ at a pulse length of 200 ns^23^. One of the important design considerations is the value of S_c_, which is maximum around the iris.

**Figure 3:**
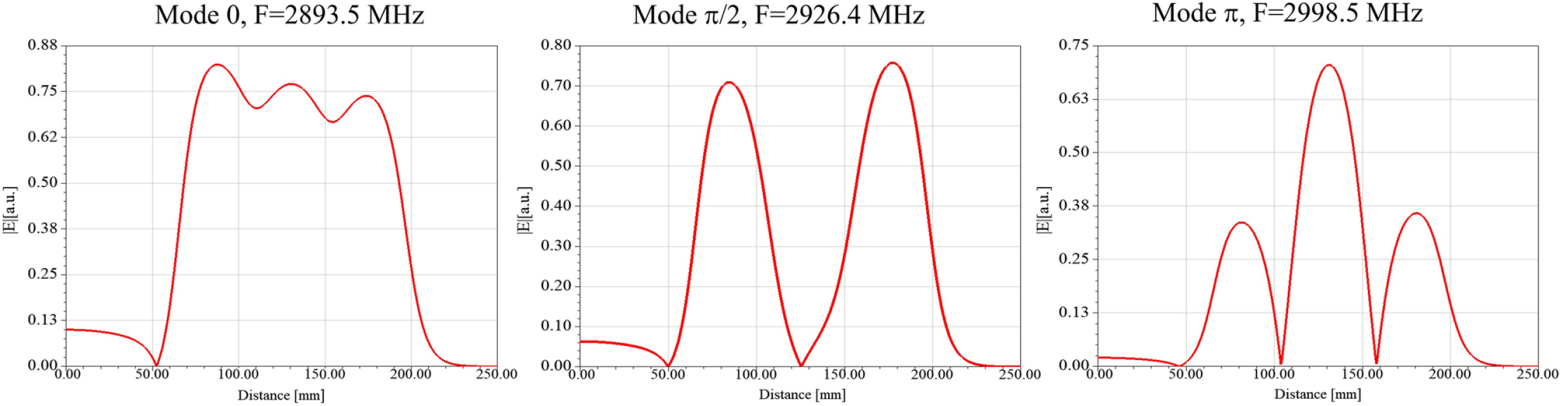
Axial electric field profile for the 3 modes.

The cavities were simulated in Ansys HFSS electromagnetic analysis software. To achieve the highest field gradient in the middle cell, a π mode was selected. By choosing an appropriate radius, π mode resonant frequency was set to 2998.5 MHz. Then, by changing a_2_ and a_3_, the axial electric field in the middle cell was adjusted to be about 2 times greater than that of the surrounding cells. Changing the iris radius led to a change in frequency. By changing the radius of the cells, π mode resonant frequency was re-set to 2998.5 MHz. After adjusting the axial electric field profile, the coupling between the cylindrical waveguide and the cavities was tuned by changing the iris of the first cell. Figure 3 shows the axial electric field profile for the three modes after tuning.

**Figure 4:**
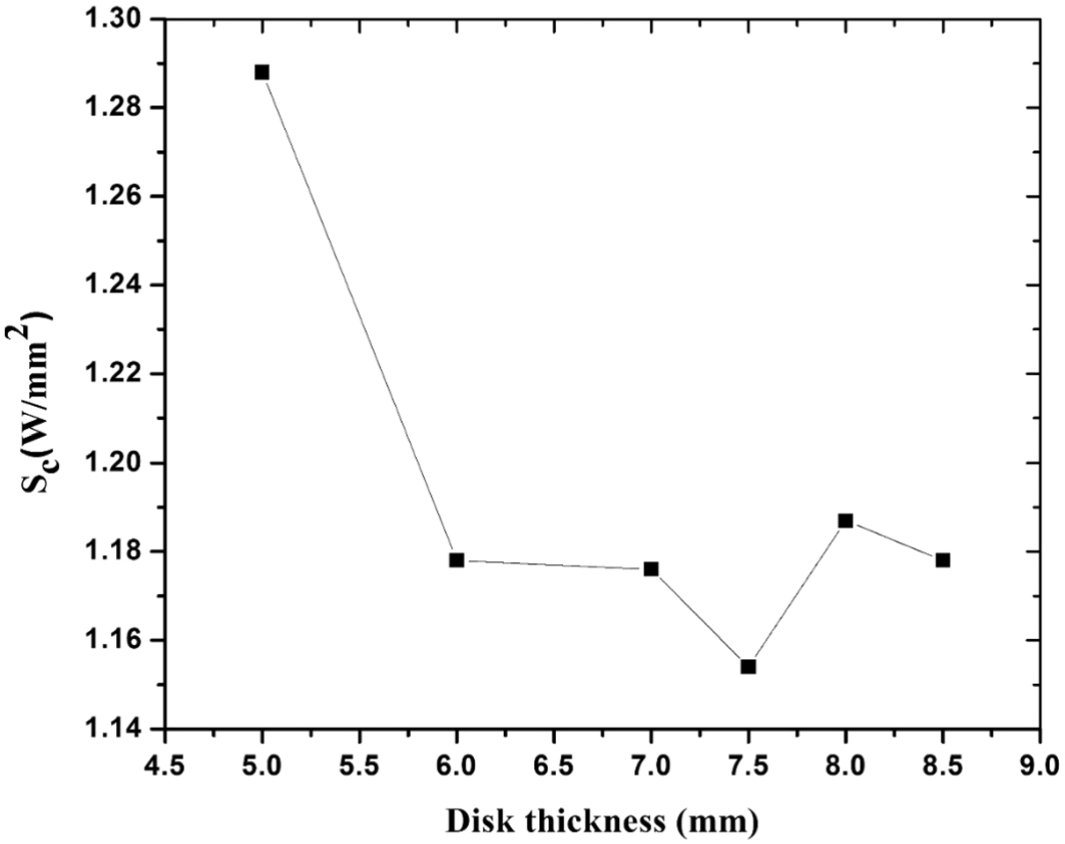
S_c_ values for different thicknesses of the disk for 1 J stored energy.

Since the value of S_c_ is highest at the irises, the iris curvature is important and it must be optimized. For this purpose, S_c_ was computed in the HFSS model and its value was calculated for different disk thicknesses, as shown in Fig. 4. The lowest value of S_c_ occurs for thicknesses between 6 and 8 mm, so finally a thickness 7.5 mm was selected. The resonant frequency changed slightly for different thicknesses, so the cavity was tuned to 2998.5 MHz again with the change of other parameters.

**Figure 5:**
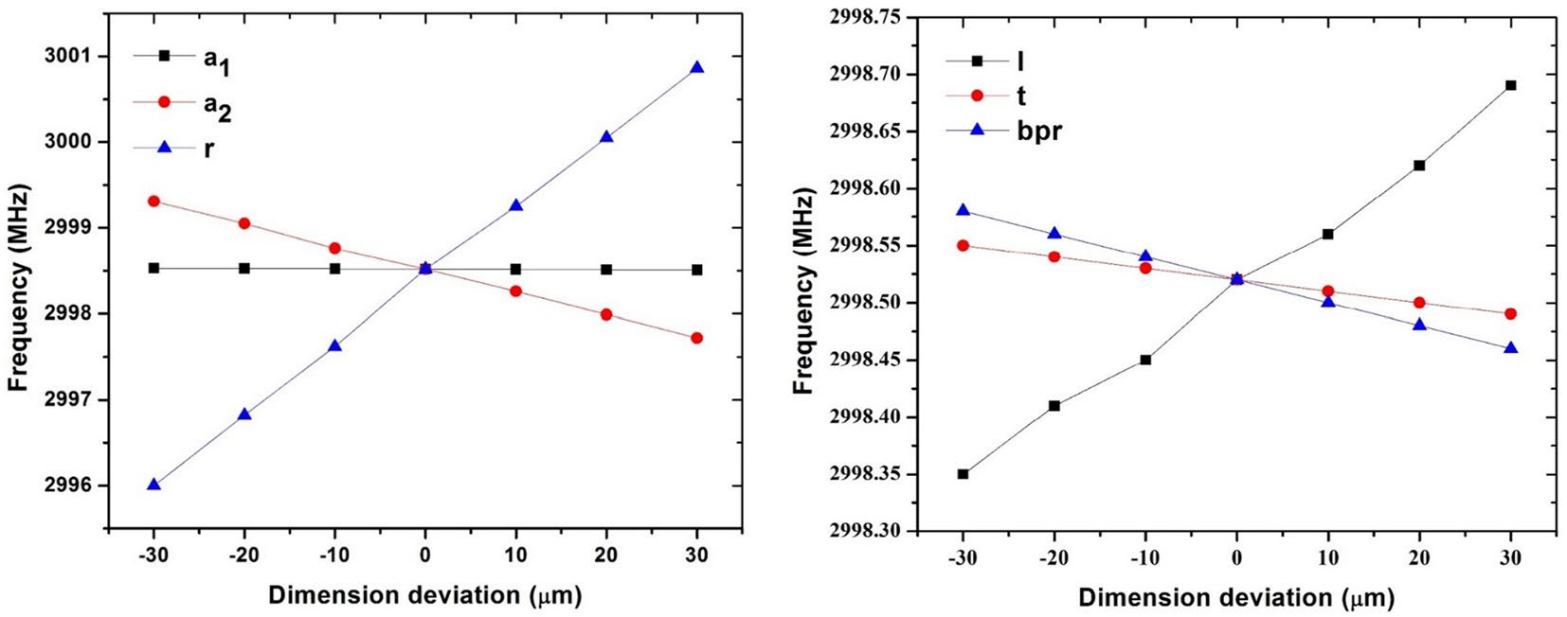
The effect of dimensional tolerance on the resonant frequency.

Table 1 and Fig. 5 show the final cavity dimensions and the effect of their errors on the resonant frequency, respectively. Because components deform in the shrink-fit method, it is important to investigate dimensional tolerances. The effect of dimensional tolerance on the resonant frequency is investigated by dimension changes of $\pm 10\,{\upmu}{\mathrm{m}}$, $\pm 20\,\upmu{\mathrm{m}}$ and $\pm 30\,\upmu{\mathrm{m}}$. After the construction, cavities can be tuned operation using four dimple tuners in each cell’s outer wall. The change in dimensions due to deformation must be such that the effect can be compensated by tuning. For S-band cavities with dimensions close to our design, it is possible to tune 3 MHz for each cell^15^. Therefore, the amount of frequency change due to the dimensional tolerances corresponding to less than $\pm 30\,\upmu{\mathrm{m}}$ can be compensated by dimple tuners. Also, the machining precision of the parts and the maximum deformation resulting from the shrink-fit method should not exceed this amount.

**Table 1:** Final dimensions of cavity’s parameters.

Parameter	r_1_ = r_2_ = r_3_ = r_4_	a_1_	a_2_	a_3_	bpr	l	t
Value (mm)	41.00	13.10	17.67	17.67	13.83	37.50	7.50

**Figure 6:**
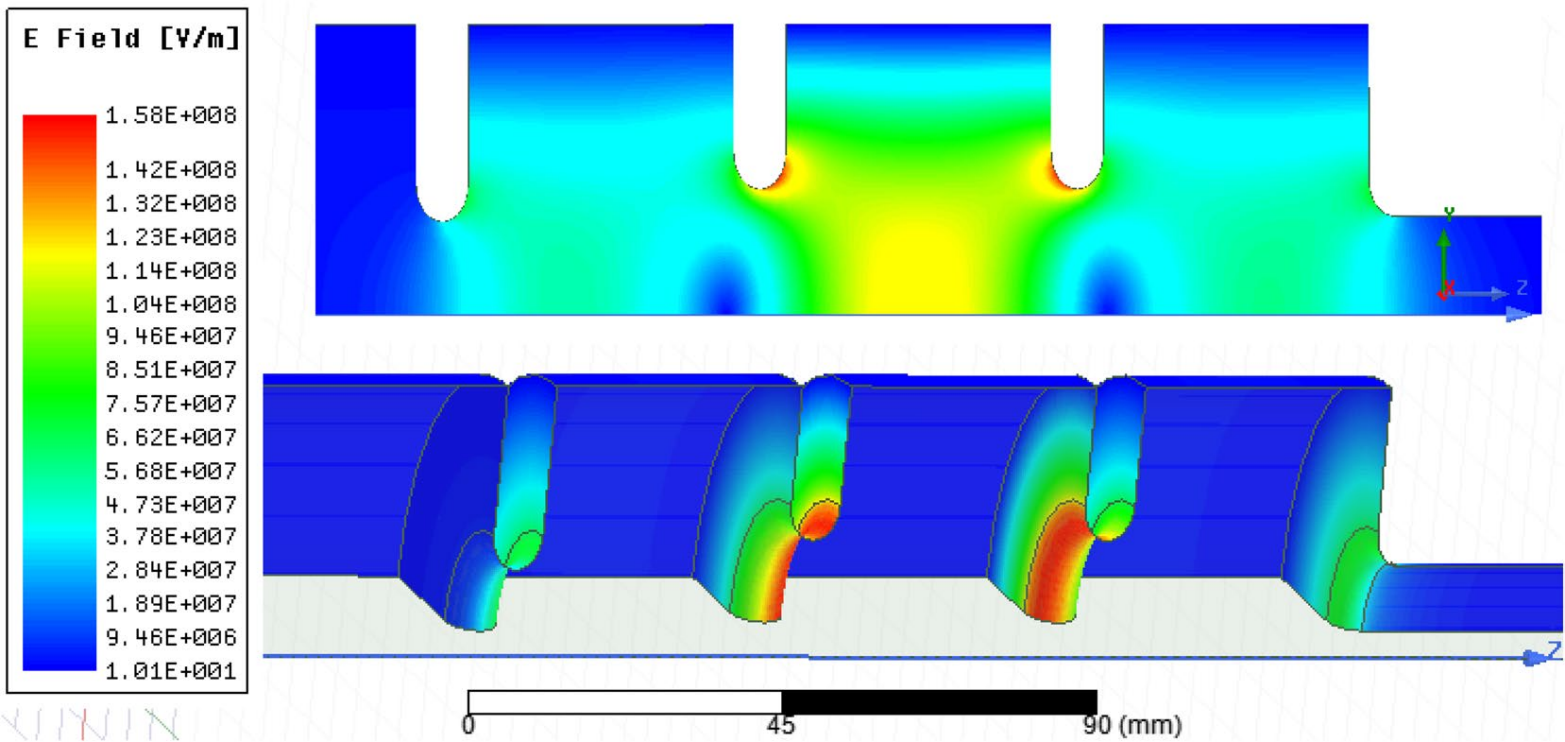
Electric field for 8 MW input power.

**Figure 7:**
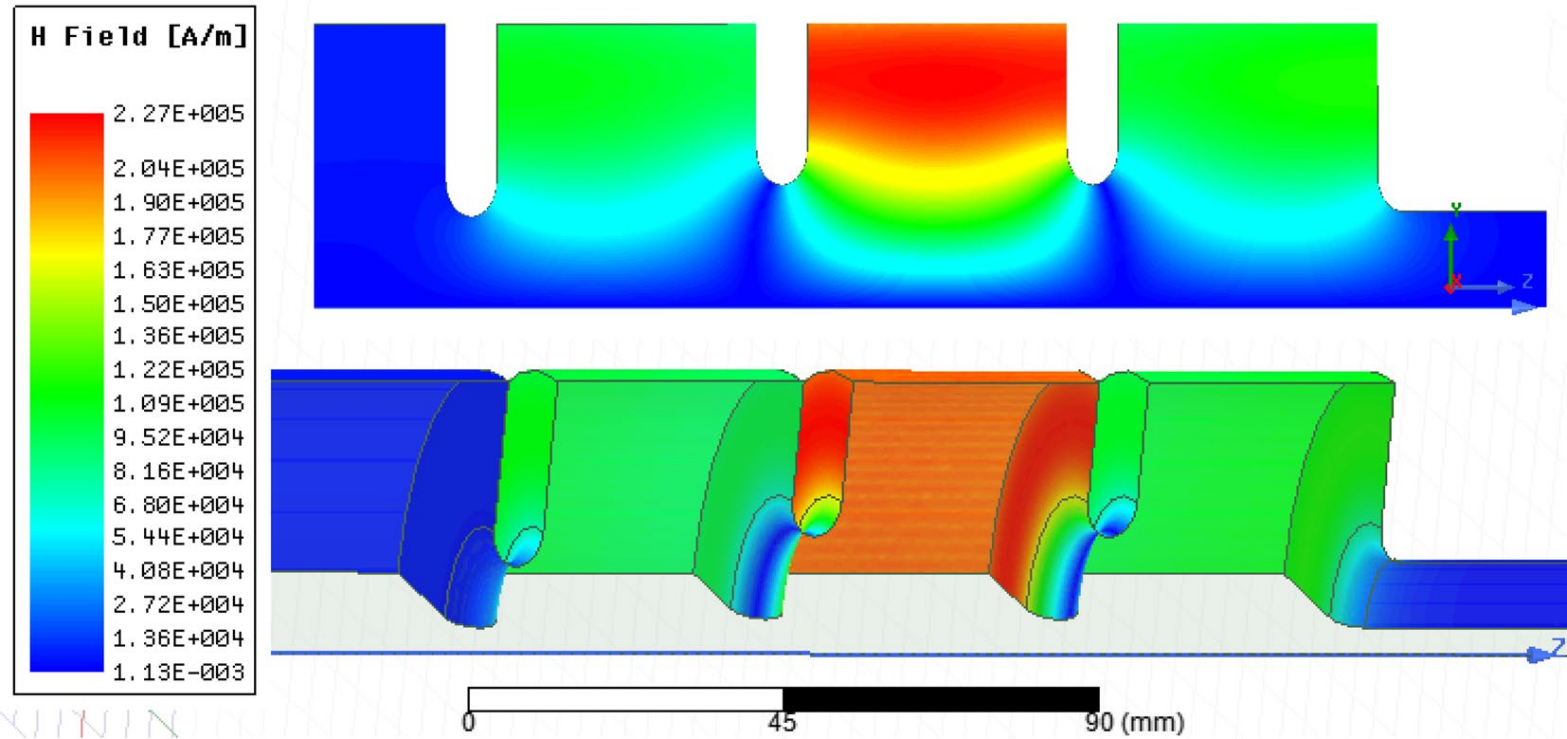
Magnetic field for 8 MW input power.

**Figure 8:**
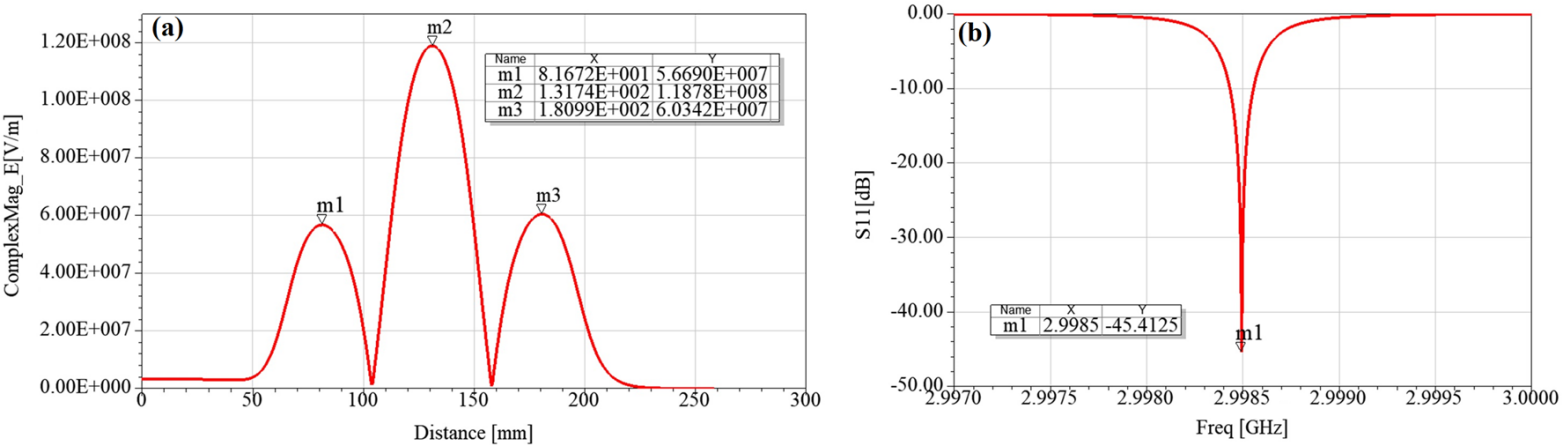
Axial electric field (**a**), and reflection coefficient (**b**) for 8 MW input power.

The electric and magnetic fields for 8 MW input power are shown in Figs. 6 and 7, respectively. The maximum surface electric field is 158 MV/m while the maximum magnetic field is 227 kA/m. Figure 8 shows the axial electric field and reflection coefficient. The maximum axial field at 8 MW is equal to 118 MV/m in the middle cell, while in the first and third cells the maximum axial field are 56 and 60 MV/m, respectively. The accelerating gradient which is the average axial electric field multiplied by transit-time factor^24^ reaches 60 MV/m at 8 MW where the transit-time-factor is 0.8. The S_11_ parameter shows a peak of − 45 dB at the resonant frequency of 2998.5 MHz.

**Figure 9:**
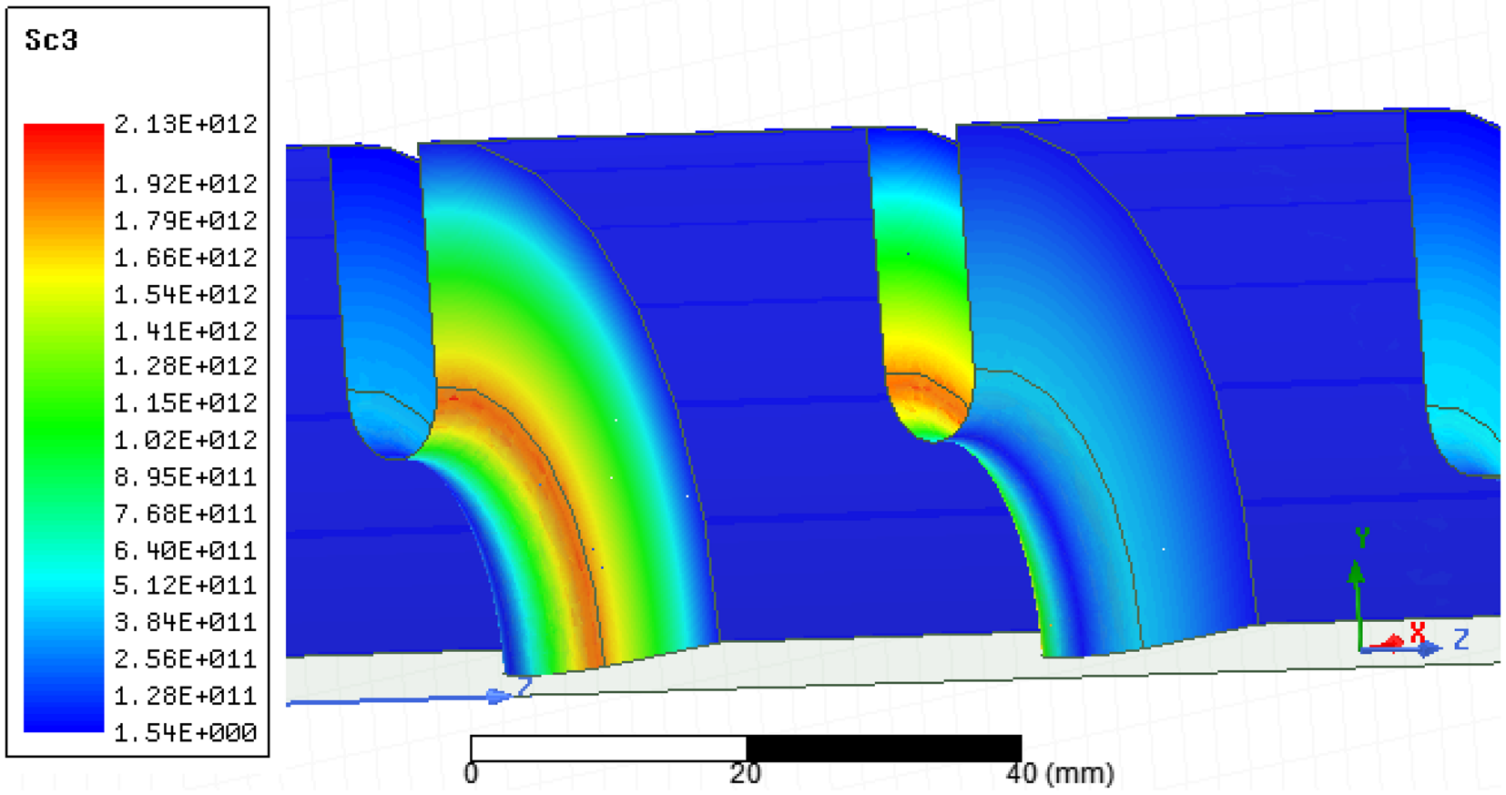
The modified Poynting vector (Sc) parameter for 8 MW input power.

S_c_ quantity for 8 MW input power is shown in Fig. 9. The maximum value of this quantity is 2.13 ${\mathrm{W}}/\upmu {\mathrm{m}}^{2}$. By rescaling CLIC experimental data ($5\,{\mathrm{W}}/\upmu {\mathrm{m}}^{2}$, 200 ns) using Eq. (2)^25^, the pulse length can be increased up to 2 μs for BDR less than ${10}^{-6}\,{\mathrm{bpp}}/{\mathrm{m}}$ at 8 MW input power.

2 $${S}_{c}^{8}{t}_{p}^{3}/BDR={\mathrm{const}}$$

A comparison was made of the fields which could be using different RF generators. The IPM electron linear accelerator laboratory in Iran has an RF test stand at frequency 2998.5 MHz based on 2 MW, KS237 klystron. The accelerating gradient and modified Poynting vector for this power are 29 MV/m and 0.53 ${\mathrm{W}}/\upmu {\mathrm{m}}^{2}$ respectively. Using Eq. (1), the pulse length can be increased up to 80 μs for BDR less than ${10}^{-6}\,{\mathrm{bpp}}/{\mathrm{m}}$ at 2 MW input power. But this test stand can also provide the mentioned power in 10 µs pulse length and 125 Hz repetition rate. Alternatively, the 8 MW klystron common in many medical accelerators which similar generator could be used for testing the cavity. Details of the simulations performed for this power are summarized in Table 2. The S-box laboratory at CERN has a maximum power of 43 MW, however only 15 MW power at a pulse length of 350 ns, would be required for accelerating gradient of 82 MV/m. Table 2 summarizes the results for the various powers. It should be noted that the filling time of the designed cavity is about 0.4 μs, however, the multi-cell cavity, which is the ultimate goal of this study, it is necessary to consider the longer filling time.

**Table 2:** The investigation of using different RF power for testing the designed cavity based on simulation results.

Input power	2 MW–10 µs (IPM RF test stand)	8 MW–6 µs	15 MW–1 µs (S-box CERN)
Max axial E (MV/m)	59	118	163
Average axial E (MV/m) in middle cell	37	75	103
Accelerating gradient (MV/m) in middle cell	29	60	82
Max surface E (MV/m)	79	157	217
Max surface B (kA/m)	113	226	312
Max S_c_	0.53	2.13	4
BDR*	1.99 × 10^–9^	2.9 × 10^–5^	2.1 × 10^–5^
Max pulse length for BDR ${<10}^{-6}\,{\mathrm{bpp}}/{\mathrm{m}}$*	80 µs	2 µs	350 ns

*By rescaling CLIC experimental data ($5\,{\mathrm{ W}}/\upmu {\mathrm{m}}^{2}$, 200 ns).

## Multiphysics investigation of the assembly method

For the construction of cavity using the shrink-fit method, the diameter of the disks is first reduced by cooling them in liquid nitrogen and then placing them inside the cylindrical waveguide. Their diameter increases as they warm to ambient temperature press against the inner surface of the cylindrical waveguide ensuring a good contact for RF currents. For a good contact it is necessary that the radius of the disks be larger than the inner radius of the cylindrical waveguide. On the other hand, the difference in diameter should not be too large so as to cause undesirable deformations. This means that the effect of the pressure of the discs on the cavity must be investigated and the resonant frequency change must be obtained so the dimensions can be pre-compensated for this effect.

**Figure 10:**
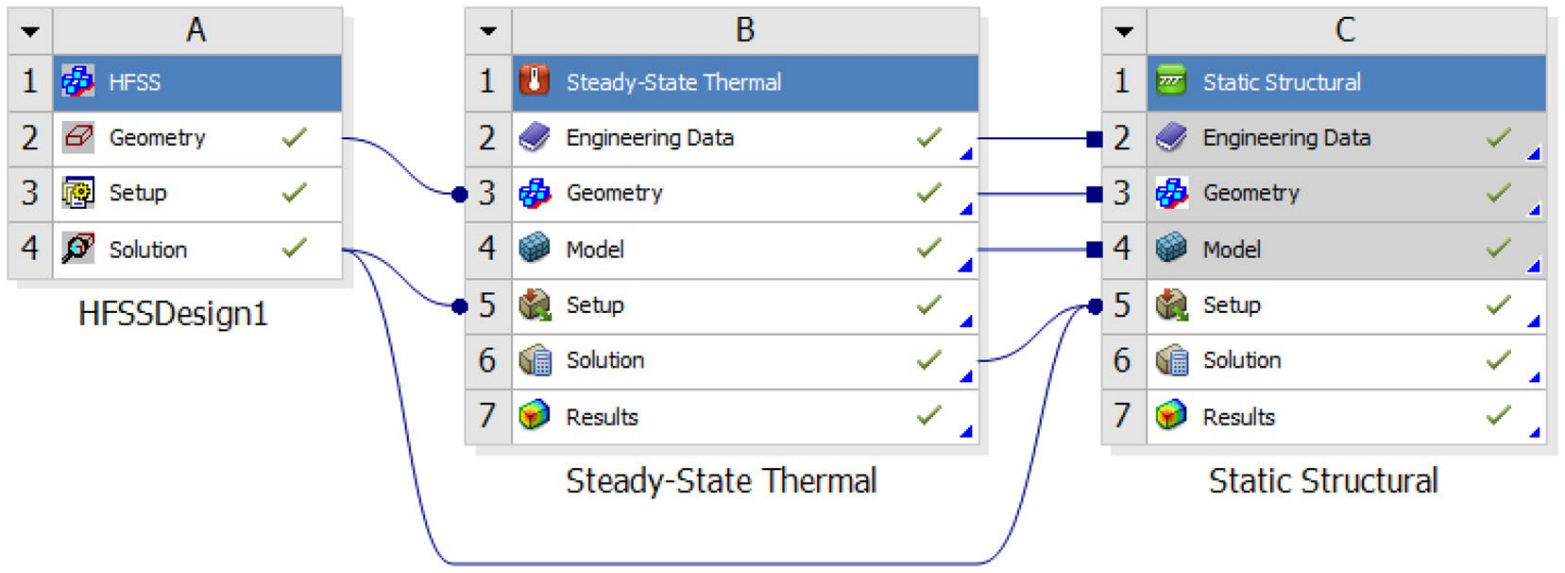
Coupling HFSS, steady state thermal (SST) and static structural in Ansys workbench for obtaining the frequency change.

For this purpose, a multi-physics study was performed by coupling mechanical and electromagnetic analysis software. The modules of Ansys software, steady-state thermal (SST) and static structural (SS), were used to calculate the amount of deformation in structure. At first, the radius of the disks was calculated at liquid nitrogen temperature. Then the temperature in which the radius of the disk is equal to the internal radius of the cylindrical waveguide (contact temperature), was calculated theoretically and the result was verified by Ansys simulation. For obtaining total deformation of the cavity, the complete set of discs and cylindrical waveguide were simulated from the contact temperature to the environment temperature. After obtaining deformed geometry, the frequency change was obtained by coupling SST and SS module with HFSS^26^. Figure 10 shows the process of coupling HFSS, steady state thermal (SST) and static structural in Ansys workbench for obtaining the frequency change.

The simulation results for disks with different radii showed that the difference in diameter of less than 100 µm is acceptable. On the other hand, 4 μm machining precision was considered. Accordingly, it is necessary that the radius difference be greater than twice the probable error (8 μm). Therefore, radius differences of 10, 20 and 30 μm were selected for further studies.

**Figure 11:**
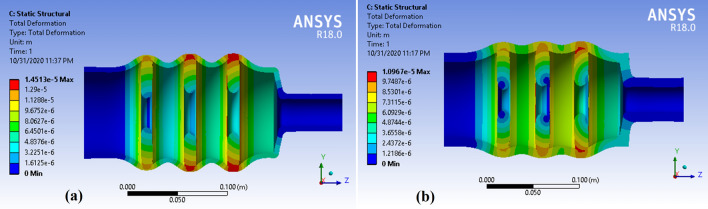
Total deformation of the cavities for two wall thicknesses of the cavity, **a** 5 mm and **b** 10 mm.

Table 3 shows the maximum deformation of cavity for different disk sizes; 10, 20 and 30 µm larger than the inner radius of the cylindrical waveguide. The thickness of the cylindrical waveguide in this step is 5 mm. The outer radius at − 195 °C and the contact temperature for disks with different iris a_1_ and a_2_ were reported too. According to the results of Table 3, considering the appropriate safe margin in machining precision and minimizing the amount of deformation, 41 mm + 20 µm radius were found to be more appropriate. The calculations in Table 3 were performed for a thickness of 5 mm for cylindrical waveguide. After selecting the appropriate radius of disk, the effect of different thicknesses of cylindrical waveguide on deformation of cavity was calculated. Figure 11 shows the total deformation of the cavity for two thicknesses of cylindrical waveguide, 5 and 10 mm, where the total deformation was shown 1000 times larger.

**Table 3:** Maximum deformation of cavity for different disk size for 5 mm cylindrical waveguide thickness.

Outer radius of disks	Outer radius in − 195 °C		Touch temperature (°C)		Maximum deformation $(\upmu {\mathrm{m}})$
	Disk a1	Disk a2, a3	Disk a1	Disk a2, a3	
41 mm + 10 µm	40.931	40.917	12	10	6
41 mm + 20 µm	40.935	40.922	0	− 5	14.5
41 mm + 30 µm	40.940	40.927	− 13	− 19	22

The maximum total deformation of cavity and frequency changes for different cylindrical waveguide thickness are summarized in Table 4. With increasing cavity thickness the deformation decreases. At a thickness of 25 mm, the changes are small in compared with 20 mm. Therefore, it is not necessary to increase the thickness more than 20 mm. For 41 mm + 20 µm radius of disks and 20 mm cylindrical waveguide thickness, the maximum deformation in the cavity is 7.8 μm. Coupling the SST and SS module with HFSS shows that the amount of resonant frequency changes in this case is 512 kHz, which can be compensated by dimple tuners.

**Table 4:** Maximum deformation of cavity for different cylindrical waveguide thickness.

Cylindrical waveguide thickness	2.5 mm	5 mm	7.5 mm	10 mm	15 mm	20 mm	25 mm
Maximum total deformation $(\upmu {\mathrm{m}})$	17.1	14.5	12.1	10.9	9	7.8	7.2
Resonant frequency change (kHz)	574	570	563	559	551	512	431

As mentioned in “Description of the shrink-fit method for the construction of high gradient cavities” section, another important issue for the shrink-fit method is how to precisely maintain the position of the discs during the assembly process. For this purpose, it was necessary to design and use a dedicated jig. The placement accuracy of the discs during the assembly process depends on the model of designed fixture and its material.

Two fixture methods were studied during this project. The first was to insert the disks one at a time in the desired location inside the cylindrical waveguide, taking the time for each to expand into place before placing the subsequent disk^16^. Although this method has good placement accuracy and is possible for small number of cavities, however, the more cavities, the harder it is to implement. The second was to use a fixture to place the discs in cylindrical waveguide at the same time (Fig. 1). Using this method, it is necessary to consider the reduction of distances between disks at liquid nitrogen temperature. For this purpose, the material of the fixture should be made from a metal with a small coefficient of thermal expansion.

The distance between the discs becomes smaller during the fixture shrinkage process. It is necessary that at the temperature at which the disks touch (Table 3), the distance between the disks is equal to the designed distance ($l$ in Table 1).

This means that the distance between the disks on the fixture at ambient temperature is compensated to be large enough to reach the designed value at touch temperature. In this case, the distance between the disks on the fixture at ambient temperature (before being placed in liquid nitrogen) is given by ${l}^{{\prime}}=l+l\alpha \Delta T$, where $l$ is the distance between the disks in the cavity design, $\alpha$ is coefficient of thermal expansion of the fixture and $\Delta T$ is difference between temperature at which the disks touch to ambient temperature. Steel was chosen for the fixture material because it has a lower coefficient of thermal expansion than other common metals. For steel, the required distance between the disks was be ${l}^{{\prime}}\approx 37.5\,{\mathrm{mm}}+15\,\upmu {\mathrm{m}}$.

The effect of the dimensional tolerance on the resonant frequency in Fig. 5 shows that if we do not include this difference in the fixture design, with a change of 15 μm in $l$, the resonant frequency change will be about 100 MHz, which is much smaller than the frequency change created by the radial expansion and it is compensable by tuning. Springs were also used to provide elasticity during the disc release process. This reduced stress and subsequent change in longitudinal dimensions. Reference^15^ provides more details on the proposed fixture.

## Experimental investigation

**Figure 12:**
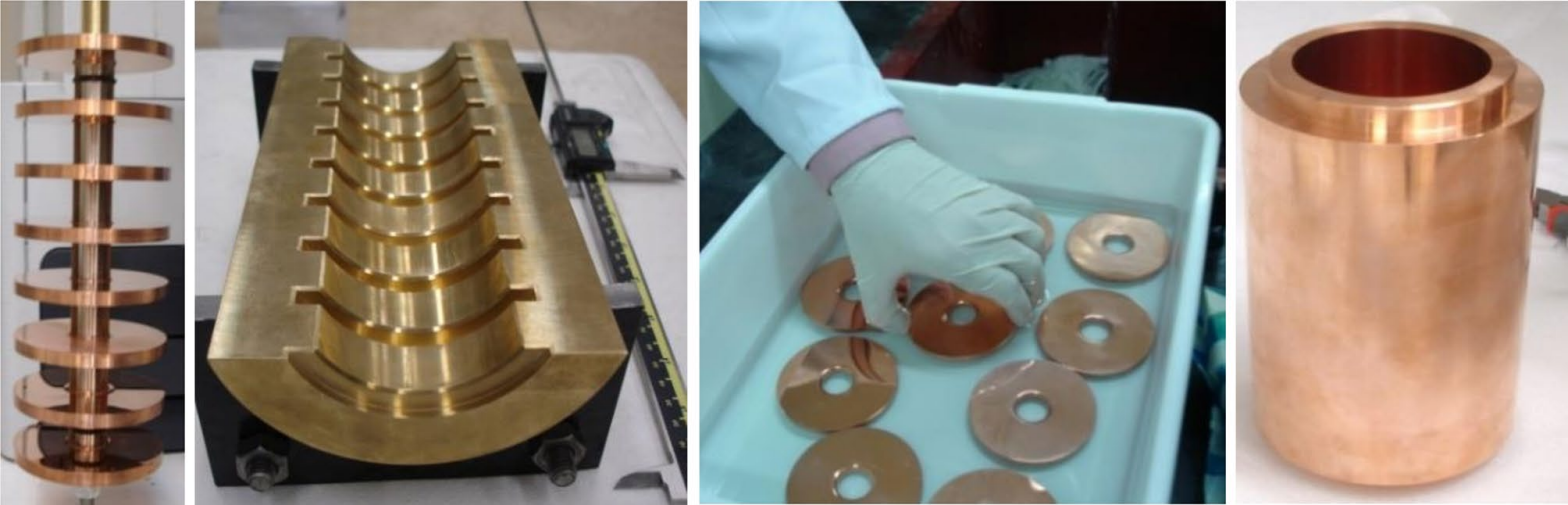
Constructed components of the cavity.

**Figure 13:**
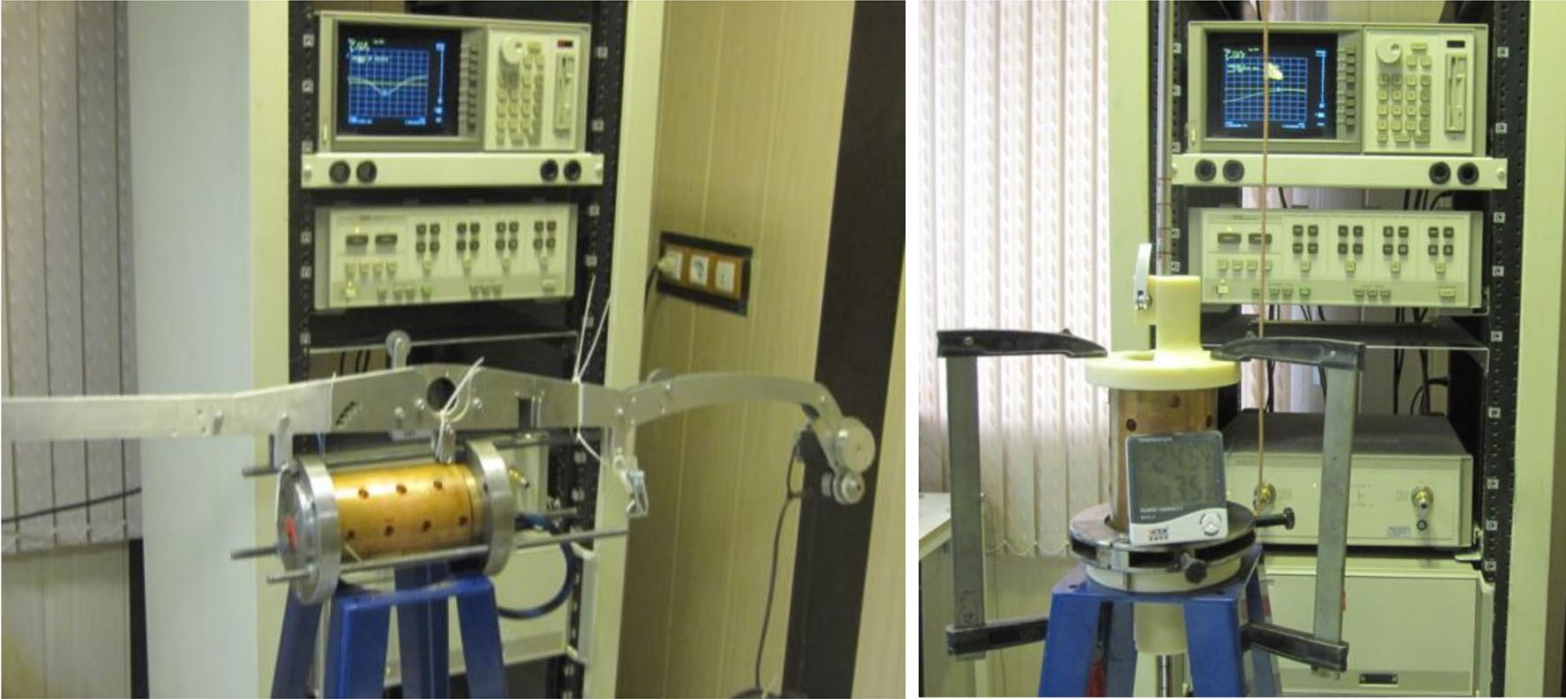
Bead-pull measurement and tuning setup.

Before construction of the 3-cell designed cavity, to ensure the feasibility of construction by shrink-fit method an eight cell cavity was produced. This section provides experimental details of its construction and RF measurement. The cavity consists of a cylindrical waveguide loaded with 8 disks close to the dimensions of the designed cavity in “Radiofrequency design” section. Figure 12 shows the machined components of this cavity. Based on the simulation results of “Multiphysics investigation of the assembly method” section, 20 mm thickness of the cylindrical waveguide and 20 µm radius difference between disk and cylindrical waveguide radius were considered for cavity. For measurements, two caps on which there are appropriate holes for placing antenna were placed at the beginning and the end of the structure. The length of the first and the last cells was half of the others; thus, there are 9 cells in the structure. The investigation includes measuring the resonant frequency of each cell and on-axis electric field by Slater method before and after tuning the cavity and comparing them with the designed value. After measuring the field and frequency of each cell, the cavity was tuned by the dimples in each cell. Three dimples 120-degree apart were used for tuning each cell. The resonant frequency of each cell and the axial field profile were measured again after tuning. Figure 13 shows the bead-pull measurement and tuning setup.

Table 5 shows the difference between the design frequency and the measured one, before and after tuning the $\pi /2$ mode in seven middle cells. The difference between the measured resonant frequency and the design value is due to three factors: 1- Deformation of the cavity during the assembly process by shrink-fit method, which maximum value was obtained in the simulations of previous section. 2- Differences in dimensions of components with design values due to machining precision. 3- The difference in the location of the disks in the cylindrical waveguide with the design values due to the assembling accuracy. After tuning the cavity, the average difference decreased from 543 to 122 kHz.

**Table 5:** Difference between the design frequency and measured one, before and after tuning for π/2 mode.

Cell number	1	2	3	4	5	6	7	Average
Before tuning (kHz)	310	410	300	980	1100	400	300	543
After tuning (kHz)	50	200	20	260	130	80	120	122

**Figure 14:**
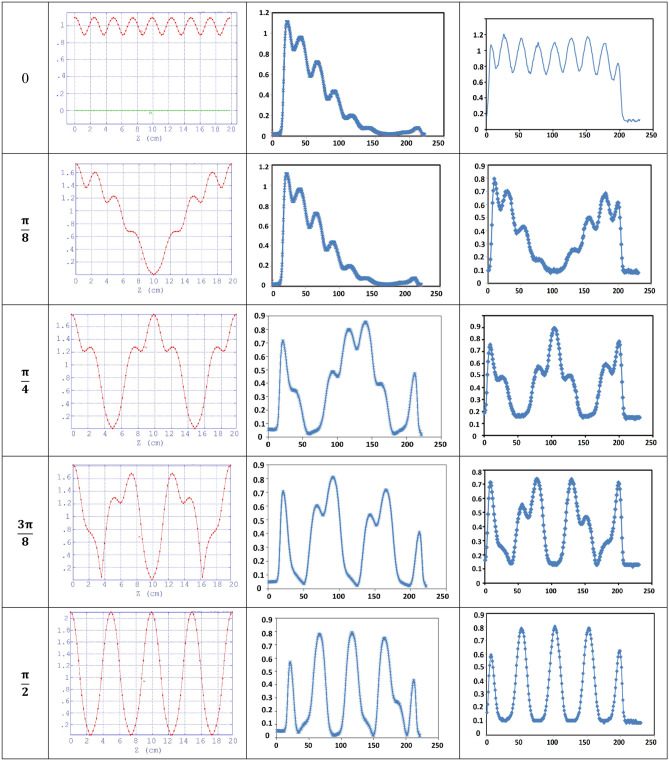

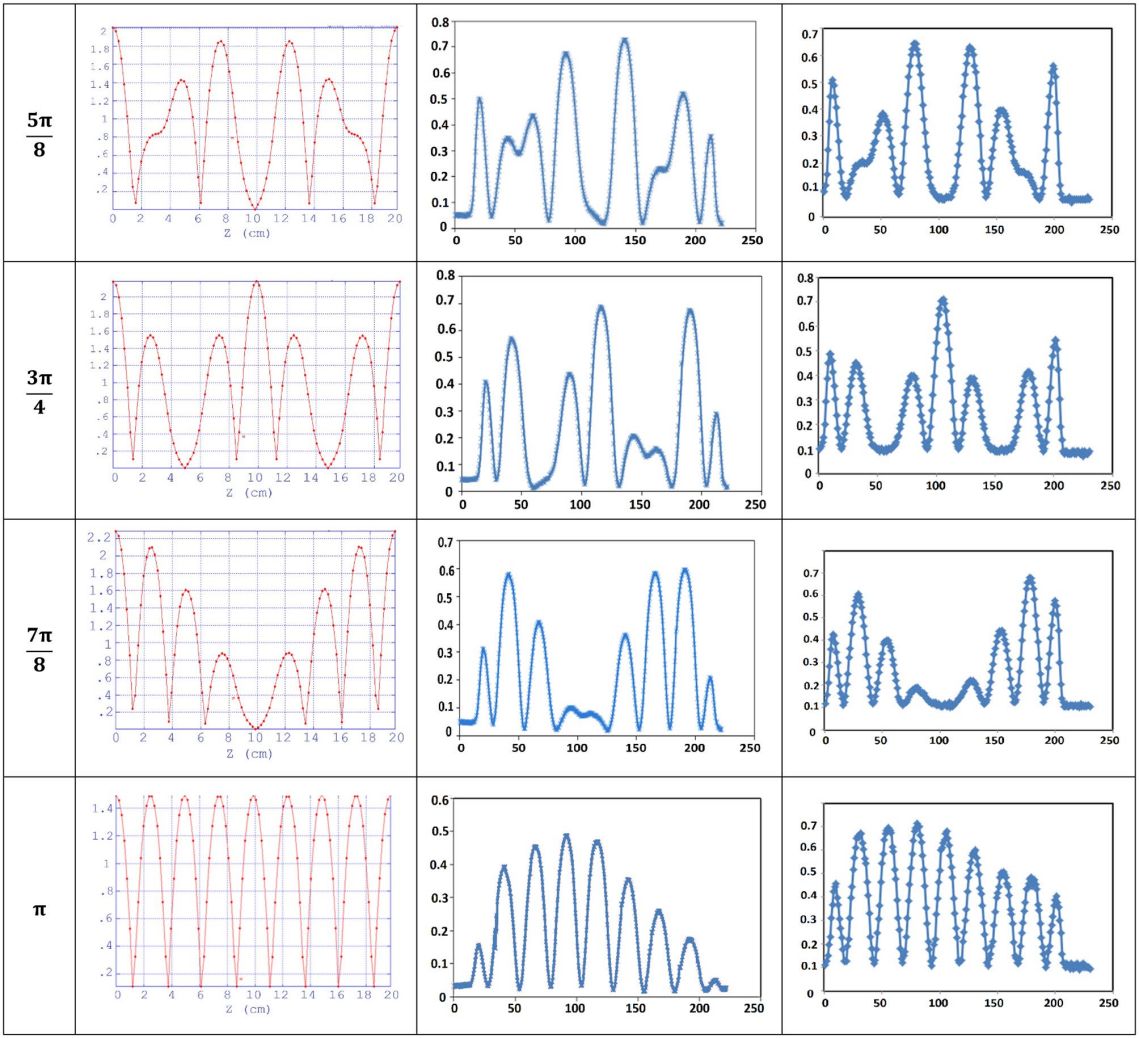
On-axis electric field for different modes (0–π from up to down with step π/8). Left: superfish simulation, middle: before tuning, right: after tuning.

Figure 14 shows the on-axis electric field of the cavity before and after tuning for different modes. The normalized electric field resulting from a Superfish code simulation is also illustrated. As shown in Fig. 12, change in electric field profile at modes are consistent with the expected results. This consistency implies that the disks are placed precisely in the cylindrical waveguide and confirms the mechanical validity of shrink-fit construction method. As mentioned in “Description of the shrink-fit method for the construction of high gradient cavities” section, the design and construction of buncher and 24-cell cavity at IPM and performing high-power tests showed an average energy of 4 MeV with 2 MW RF input power^18,19,21^ confirms the high-power feasibility of the shrink-fit construction method.

## Conclusion

This paper describes the use of a shrink-fit method for the construction of high gradient cavities and describes a detailed study of the important aspects of the design of a three-cell test cavity. For the radio frequency design, the axial electric field is adjusted so that the field in the middle cell is twice of the field in the surrounding cells. The designs showed an accelerating gradient of 29, 60 and 82 MV/m achievable for 2, 8 and 15 MW input power respectively. Also, the study of the modified Poynting vector showed that for mentioned input powers, the breakdown rate less than ${10}^{-6}\,{\mathrm{bpp}}/{\mathrm{m}}$ is accessible for maximum pulse length 80 µs, 2 µs and 350 ns.

Using mechanical analysis software and by multi-physics studies, the shrink-fit method was evaluated for the construction high gradient cavities. 3-cell cavity were simulated using Ansys software and the value of the external radius of the disks and the wall thickness of the cavity were optimized to obtain minimum deformation in the structure.

For the desired geometry of this project, the external radius of disks and cylindrical waveguide thickness are $41\,{\mathrm{mm}}+20\,\upmu{\mathrm{m}}$ and 20 mm, respectively. By coupling the statistical structure module and HFSS, the amount of resonant frequency changes due to shrink-fit construction method was calculated. This value is less than 600 kHz for designed cavity in this article, which can be compensated by dimple tuners. Also, to ensure the feasibility of construction by shrink-fit method, a sample cavity was constructed and cold tests was performed. Bead-pull measurement showed that electric field profile at different modes are consistent with the simulation results. Following the project, construction of the designed 3-cell cavity and high gradient tests are underway.
